# Neck/shoulder pain in adolescents is not related to the level or nature of self-reported physical activity or type of sedentary activity in an Australian pregnancy cohort

**DOI:** 10.1186/1471-2474-10-87

**Published:** 2009-07-20

**Authors:** Andrew M Briggs, Leon M Straker, Natasha L Bear, Anne J Smith

**Affiliations:** 1 School of Physiotherapy and Curtin Health Innovation Research Institute, Curtin University of Technology, Perth, Australia; 2 Telethon Institute for Child Health Research, Perth, Australia; 3 Department of Physiotherapy, Princess Margaret Hospital for Children, Perth, Australia

## Abstract

**Background:**

An inconsistent relationship between physical activity and neck/shoulder pain (NSP) in adolescents has been reported in the literature. Earlier studies may be limited by not assessing physical activity in sufficient detail. The aim of this study was to comprehensively examine the association between NSP and the level and nature of physical activity, and type of sedentary activity in adolescents.

**Methods:**

A cross-sectional analysis using data from 924 adolescents in the Western Australian Pregnancy Cohort (RAINE) study was performed. Complete data were available for 643 adolescents (54.6% female) at the 14-year follow-up. Physical activity was measured using a detailed self-report electronic activity diary requiring participants to input details of all physical activities over the day in segments of 5 minutes for a one-week period. Physical activity *levels *were categorised as: sedentary, light, moderate, or vigorous based on metabolic energy equivalents. *Nature *of activity was determined by assigning each activity to categories based on the amount of movement (static/dynamic) and the main posture assumed for the activity (standing/sitting/lying). *Type of sedentary activity *was characterised by exposure time to watching TV, using a computer, and reading. Logistic regression was used to explore the association between NSP and activity.

**Results:**

Females reported a higher prevalence of lifetime, 1-month and chronic NSP than males (50.9 vs 41.7%, 34.1 vs 23.5%, and 9.2 vs 6.2% respectively). No consistent, dose-response relationship was found between NSP and the level, nature, and type of physical activity.

**Conclusion:**

Self-reported one month and lifetime NSP prevalence in adolescents is *not *related to the level or intensity of physical activity or the type of sedentary activity over a one week period.

## Background

Neck/shoulder pain (NSP) is common among the general population with the 12 month prevalence ranging from 12–72% in adults [1] and often results in a significant burden on workforce productivity [2]. The impact and correlates of NSP are relatively well established in adults [1], with both physical and psychosocial risk factors reported [3,4]. Yet in adolescents, the correlates for NSP are less certain. Of concern is that the prevalence of NSP is high among adolescents [1,5] and appears to be increasing [6], raising a stimulus for targeted research.

It is important to investigate the aetiology of adolescent spinal pain since prospective studies suggest back pain experienced in youth is a predictor for back pain in adulthood [7-9]. It is likely that this same trajectory is also applicable to NSP, and there is some evidence to support this suggestion [10-13]. Moreover, the impact of NSP in young people is not trivial, with NSP interfering with school and leisure activities [14]. Neck pain is reported to be the most common musculoskeletal pain site in adolescents [14-16] and the most common site for persistent symptoms [17]. Notably, research examining modifiable factors (specifically physical activity) associated with NSP was identified as a major research priority by the Bone and Joint Decade 2000–2010 Task Force on Neck Pain and it's Associated Disorders [18,19].

Physical activity is an important determinant of musculoskeletal and general health across the lifespan [20]. Of concern is that participation in physical activity by adolescents appears to be decreasing [21] while participation in sedentary behaviours such as computer and electronic game use is increasing [22]. The incidence of chronic health conditions associated with decreased physical activity, such as obesity, is increasing rapidly in Australia [23] and other nations [24,25] in parallel with adolescent spinal pain conditions [6]. There is evidence that these lifestyle changes have implications for musculoskeletal health. For example, adolescent and young-adult neck and back pain has been related to increased time using information technology [6,10,26], while prospective data suggest that physical inactivity in young adulthood is associated with chronic musculoskeletal complaints in later life [27].

A recent systematic review reported no consistent relationship between BMI and neck pain, suggesting that obesity-related physical inactivity is not associated with neck pain among the general population [1]. Moreover, there was no consistent relationship between participation in exercise and sporting activities and neck pain [1]. In adolescents however, there is some evidence to suggest that NSP is mediated by the intensity of activity, with adolescents who are very active and those who are very inactive reporting a greater prevalence of NSP. Therefore, the relationship between NSP and activity may differ with the level of activity [26,28-30]. Nonetheless, in other studies no association has been reported between adolescent NSP and physical activity [14-16,31-34].

The discrepancies reported in the literature may be attributable to differences in the definitions of and measurement approaches for pain and activity. Critical weaknesses of earlier investigations include a reliance on crude self-reports for physical activity data, which are known to correlate poorly with objective measures of physical activity [35], limited data reported, inconsistencies in classifications with respect to activity patterns, variability in the definitions of physical activity, and cut points for categorisation of physical activity. For example collecting information regarding activity or inactivity in isolation and considering only activity during leisure time rather than across the entire day does not give an accurate picture of habitual activity levels. Moreover, different types of activity [13,29,34] and inactivity [26] may relate to NSP differently, reflecting unique biomechanical and cognitive characteristics associated with different activities. This highlights the importance of examining the relationship between types of activity and NSP rather than considering activity as a homogenous construct.

The aim of this study was to further clarify the relationship between adolescent NSP and physical activity using a detailed characterisation of physical activity and inactivity. Our objective was to broadly replicate the method and extend the findings of Auvinen et al [26] in order to compare findings between Australian and Finnish birth cohorts.

## Methods

### Participants

Data were collected from 643 adolescents (351 females, 292 males) of mean (SD) age 14.03 (0.19) years from the Western Australian Pregnancy Cohort "Raine" Study . This project began with a cohort of women attending antenatal clinics at King Edward Memorial Hospital for Women, Perth, Australia between 1989 and 1991. The children have been followed at birth, 1, 2, 3, 5, 8, 10, and 14 years of age. 2425 adolescents were eligible for the 14 year follow-up, and 1861 (76.7%) of these agreed to participate in some aspect of the follow-up. 924 adolescents (38.1% of the cohort eligible for follow-up at 14 years) participated in this study, of which complete data were available for 643 (69.6%). There were no exclusion criteria for this part of the cohort. The participant characteristics mirror those of the general Western Australian population into which they were born, except for the following characteristics: a higher prevalence of pre-term and low birth weight children, twins and triplets, and those who took longer to achieve spontaneous respiration at birth, suggesting that this cohort had higher than usual obstetric risk. The sociodemographic characteristics of the cohort families also mirrors those of the general Western Australian population, except for a lower proportion of fathers employed in managerial positions and a higher proportion of fathers employed in professional positions [36]. We therefore consider the characteristics of the cohort presented here to be generalisable to the Australian population and comparable to the Finnish birth cohort [26].

Parents/guardians provided written informed consent prior to participation. The study was approved by the Human Research Ethics Committees of Curtin University of Technology and Princess Margaret Hospital, Western Australia, in accordance with the Declaration of Helsinki 1975.

### NSP prevalence

Participants completed a laptop-based questionnaire at an assessment centre. Adolescents were asked about their lifetime, one-month, and chronic (> 3-month duration) experience of NSP, described as pain in the area of the posterior neck and upper trapezius, as diagrammatically defined by Kourinka et al. [37]. The relevant NSP questions were: "Have you ever had neck/shoulder pain?" ("yes" or "no"), "Has your neck/shoulder been painful in the last month?" ("yes" or "no"), and "Did your neck/shoulder pain last for more than 3 months?" ("yes" or "no"). Validity and reliability for the Nordic questionnaire have been established previously [37,38].

### Physical Activity characterisation

Physical activity type, duration and intensity were assessed using a self-report electronic activity diary known as Multimedia Activity Recall for Children and Adolescents (MARCA) [39]. Participants were provided with MARCA software to use on their home computer or a laptop borrowed from the research centre. Participants were instructed to input nightly the details of all activities they performed over the day, in segments of 5 minutes duration, for a week immediately following completion of the NSP questionnaire. Activities were selected from a dropdown menu of more than 250 activities. In circumstances where the physical activity performed was not listed in the drop-down menu, participants selected 'other' and typed a description of their activity. Researchers then matched this activity with the closest possible MARCA equivalent in terms of activity type. Only data sets including at least 3 weekdays and 1 weekend day were included. Data accuracy was maintained by examining obvious outliers for internal consistency. Concurrent validity (r = 0.36–0.45) and test re-test reliability (ICC = 0.88–0.94) for MARCA have been established previously in children aged 11 years and over [39].

The intensity of physical activity was calculated by assigning a metabolic energy equivalent (MET) value to each activity in MARCA. Physical activity *levels *were categorised as: sedentary activity (1 METS), light activity (>1–3 METS), moderate activity (>3–6 METS), or vigorous activity (> 6 METS), and expressed as the hours/week for each level. The MET estimates were based on the compendium or energy expenditures for youth [40] modified from widely used adult compendium [41]. The *nature *of activity was calculated by assigning each activity to categories based on the amount of movement (static, dynamic) and the main posture for the activity (standing, sitting and lying), and expressed as the hours/week. *Type of sedentary activity *was characterised by exposure time (hours/week) to watching TV, using a computer, reading, and total electronic information technology activities (TV and computer).

### Other measures

After the adolescents completed the questionnaire, basic anthropometric measures were taken (height and mass) in order to calculate body mass index (BMI kg/m^2^). Subjects were classified as overweight or obese according to their BMI using the reference standards outlined by Cole et al [42]. The overweight and obesity cut off points recommended were derived from six large nationally representative growth studies and therefore provide an internationally acceptable reference for assessing overweight and obesity in children. This method has been used previously for adolescents [16,34]. Females were classified as overweight or obese with a BMI of 23.34–28.56 and ≥28.57 respectively. Males were classified as overweight or obese with a BMI of 22.62–27.62 and ≥27.63 respectively. Information on smoking status was obtained from the questionnaire. The adolescent questionnaire included the questions "Have you ever smoked even part of a cigarette?" and "Have you smoked cigarettes in the past 12 months?" and several questions covering the number of cigarettes smoked in the past week. Subjects were categorized as non-smokers (never smoked, only a few puffs ever, or no smoking in past 12 months), occasional smokers (smoking past 12 months) or a smoker (smoking past week).

### Data analysis

Each of the continuous variables from MARCA for physical activity, nature of activity and specific sedentary activities were banded to form five groups. The majority of variables were normally distributed and subsequently grouped according to percentiles (approximately < 10^th^, 10^th^–25^th^, 25^th^–75^th^, 75^th^–90^th ^and > 90^th ^percentile) keeping reasonable values to assist with the final interpretation. Where the distribution was not normal the variables were grouped into 5 categories with the lowest category consisting of subjects with 0 hours exposure and the remaining data grouped according to percentiles (approximately < 25^th^, 25^th^–50^th^, 50^th^–75^th ^and > 75^th ^percentile), again keeping reasonable values. Independent t-tests were used to compare anthropometrics between genders while chi-square tests were used to calculate any significant difference in proportions of NSP prevalence and physical activity between genders. Logistic regression was used to explore the association between adolescent NSP and physical activity level, nature of activity and type of sedentary activity. Logistic regression models for NSP ever, NSP in the past month, and chronic NSP (pain of more than 3 months duration), and activity level, nature of activity and specific types of sedentary activities were performed. Analyses were conducted separately for males and females. The selection of the reference group for the different logistic regression models varied. For models using *activity level *and *nature *of the activity the reference group was adolescents in the middle category of exposure time. The middle category was viewed to represent moderate exposure so that the relative association of high and low exposure activity with NSP could be evaluated. For the specific *sedentary activities *(reading/computer/TV/IT) the reference groups were regarded as those who spent the least amount of time in each of the activities. This reference group selection enabled the evaluation of the association between increasing exposure to sedentary activity and NSP to be explored as it is believed that increasing sedentary activity is associated with NSP. Odds ratios and their 95% confidence intervals (CIs) were calculated and adjustments for smoking and BMI made, in keeping with earlier studies [26,43-45]. The adjusted ORs and their CIs for NSP in the past month are shown in this paper. We report in detail the 1 month prevalence here as this period has previously been identified as the most reliable recall period for adolescent spinal pain [46] and is in agreement with an international consensus group guidelines for prevalence studies of low back pain [47]. Statistical analysis was performed using Stata Version 8.2 (StatCorp, Texas). Both male andfemale samples were sufficiently large to have over 90% power to detect odds ratios of 1.5 and above at α = 0.05 (Stata Version 8.2 (StatCorp, Texas))

## Results

Table 1 reports the physical characteristics and prevalence of NSP while Table 2 reports the physical activity data for the 351 females and 292 males who participated in this study. While there was no difference in age and mass between genders, males had a significantly greater height and females had a larger BMI (p < 0.0001 and p = 0.018 respectively). The prevalence of reporting NSP ever or NSP in the past month was higher in females (p = 0.02 and p = 0.003 respectively), while there was no difference in the gender proportions reporting chronic NSP (NSP lasting for 3 months or more) (Table 1).

**Table 1:** Physical characteristics and prevalence of neck/shoulder pain in females (n = 351) and males (n = 292).

**Descriptor**	**Females**	**Males**	**p-value**
**Physical characteristics (mean, SD)**			
Age (years)	14.0 (0.19)	14.0 (0.19)	0.184
Height (cm)	162.4 (6.3)	166.4 (9.1)	<0.0001
Mass (kg)	56.7 (12.3)	57.5 (13.1)	0.458
BMI (kg/m^2^)	21.4 (4.1)	20.6 (3.7)	0.018
			
**Prevalence of neck/shoulder pain (%)**			
no neck pain	49.1	58.3	0.02
neck pain ever	50.9	41.7	0.02
neck pain in past month	34.1	23.5	0.003
chronic neck pain (> 3 month duration)	9.2	6.2	0.164

**Table 2:** Physical activity characteristics in females (n = 351) and males (n = 292) according to level of physical activity, nature of physical activity, and sedentary activity characteristics.

**Physical activity characteristic**	**Females**	**Males**	**p-value**
**Level (%)**			
			
**Sedentary activities (1 MET)**			
More than 75 hours/week	14.0	11.6	
>70–75 hours/week	32.5	33.2	
>65–70 hours/week	23.9	18.5	0.283
>60 to 65 hours/week	22.2	26.4	
60 hours/week or less	7.4	10.3	
**Light activities (>1–3 METs)**			
More than 95 hours/week	12.5	14.0	
>90–95 hours/week	16.8	14.0	
>80–90 hours/week	47.6	37.7	0.003
>70–80 hours/week	18.0	22.3	
70 hours/week or less	5.1	12.0	
**Moderate activities (>3–6 METs)**			
More than 18 hours/week	10.3	12.7	
>12–18 hours/week	18.2	13.0	
>5–12 hours/week	53.3	42.1	<0.0001
>3–5 hours/week	11.4	15.8	
3 hours/week or less	6.8	16.4	
**Vigorous activities (> 6 METs)**			
More than 8 hours/week	8.3	29.4	
>4–8 hours/week	18.2	29.4	
>2–4 hours/week	25.1	15.8	<0.0001
>0–2 hours/week	32.5	15.5	
0 hours/week	15.9	9.9	
			
**Nature (%)**			
			
**Dynamic activities**			
More than 40 hours/week	8.0	5.1	
>30–40 hours/week	19.9	18.5	
>20–30 hours/week	50.2	42.8	<0.0001
>15–20 hours/week	16.5	15.1	
15 hours/week or less	5.4	18.5	
**Static activities**			
More than 155 hours/week	1.7	8.2	
>145–155 hours/week	31.1	30.1	
>135–145 hours/week	43.0	41.8	0.002
>130–135 hours/week	10.8	10.6	
130 hours/week or less	13.4	9.3	
**Lying**			
More than 90 hours/week	9.7	9.9	
>80–90 hours/week	21.6	27.4	
>70–80 hours/week	45.9	36.0	0.157
>65–70 hours/week	14.2	17.5	
65 hours/week or less	8.6	9.2	
**Sitting**			
More than 70 hours/week	10.8	17.8	
>60–70 hours/week	26.2	31.2	
>50–60 hours/week	34.5	28.4	0.016
>40–50 hours/week	18.0	16.4	
40 hours/week or less	10.5	6.2	
**Standing**			
More than 20 hours/week	12.2	2.0	
>15–20 hours/week	24.5	9.6	<0.0001
>10–15 hours/week	40.2	29.8	
>5–10 hours/week	19.7	41.1	
5 hours/week or less	3.4	17.5	
			
**Sedentary activities (%)**			
			
**Time watching TV**			
More than 32 hours/week	7.1	13.7	
>23–32 hours/week	12.5	19.5	
>10–23 hours/week	49.9	45.6	0.001
>6–10 hours/week	19.1	12.3	
10 hours/week or less	11.4	8.9	
**Total reading time**			
More than 4 hours/week	18.5	18.5	
>2–4 hours/week	22.8	17.1	
>1–2 hours/week	17.6	17.8	0.115
>0–1 hours/week	19.7	17.5	
0 hours/week	21.4	29.1	
**Time at computer**			
More than 12 hours/week	17.1	26.0	
>6–12 hours/week	23.6	23.3	
>3–6 hours/week	23.1	19.5	0.072
>0–3 hours/week	25.1	22.9	
0 hours/week	11.7	8.2	
**Total IT time (TV + computer)**			
More than 46 hours/week	3.1	17.5	
>35–46 hours/week	10.5	19.2	
>18–35 hours/week	52.2	46.9	<0.0001
>12–18 hours/week	18.5	10.9	
12 hours/week or less	15.7	5.5	

There were statistically significant differences in the levels of light, moderate and vigorous activity between males and females (see Table 2). There were more males in the lowest volume category of light and moderate activities and more males in the highest category of vigorous activity. There were statistically significant gender differences in the levels of activity as classified by nature of activity, with more males in lowest categories of dynamic and standing activities and the highest categories of static and sitting activities (see Table 2). Statistically significant gender differences were found for the sedentary activities of watching TV and total IT time (TV plus computer).

### Level of physical activity and NSP

No statistically significant associations were found for NSP (ever, past month or chronic NSP) and light or moderate physical activities. Sedentary activities of >70–75 hours per week compared to >65–70 hours per week was found to be protective against NSP in the past month for females (OR 0.44; 95% CI 0.23–0.85) and males (OR 0.38; 95% CI 0.15–0.96) (Table 3). Sedentary activity was neither significantly related to NSP ever nor chronic NSP. No statistically significant associations were found for vigorous activity and NSP ever and NSP in the past month. In females, vigorous activity of more than 8 hours per week compared to >2–4 hours per week was found to be associated with chronic NSP (OR 7.90; 95% CI 2.36–26.39), but not in males.

**Table 3:** Logistic regression of neck/shoulder pain (NSP) in the past month and physical activity level for females (n = 351) and males (n = 292), expressed as odds ratio and 95% confidence intervals (CI)

Physical activity level	**Females**		**Males**	
	Proportion reporting NSP (%)	Adjusted^a ^OR (CI)	Proportion reporting NSP (%)	Adjusted^a ^OR (CI)
**Sedentary (1 MET)**				
More than 75 hours/week	28.6	0.64 (0.31–1.33)	26.5	0.94 (0.39–2.27)
>70–75 hours/week	21.7	*0.44 (0.23–0.85)	13.0	*0.38 (0.15–0.96)
>65–70 hours/week	38.6	1	27.8	1
>60 to 65 hours/week	46.8	1.37 (0.76–2.47)	27.0	0.96 (0.48–1.88)
60 hours/week or less	26.9	0.59 (0.23–1.53)	16.7	0.51 (0.18–1.48)
				
**Light (>1–3 METs)**				
More than 95 hours/week	36.4	1.40 (0.69–2.85)	20.0	0.79 (0.32–1.94)
>90–95 hours/week	44.1	1.79 (0.96–3.30)	25.0	1.06 (0.45–2.45)
>80–90 hours/week	28.9	1	23.8	1
>70–80 hours/week	35.5	1.35 (0.73–2.51)	24.6	1.04 (0.51–2.13)
70 hours/week or less	38.9	1.72 (0.61–4.84)	22.9	0.96 (0.38–2.38)
				
**Moderate (>3–6 METs)**				
More than 18 hours/week	41.7	1.49 (0.71–3.14)	24.3	1.14 (0.48–2.71)
>12–18 hours/week	35.5	1.09 (0.60–1.99)	21.6	0.97 (0.40–2.36)
>5–12 hours/week	34.2	1	22.1	1
>3–5 hours/week	25.0	0.66 (0.30–1.44)	22.2	1.02 (0.45–2.34)
3 hours/week or less	33.3	0.99 (0.40–2.45)	29.2	1.45 (0.68–3.10)
				
**Vigorous (> 6 METs)**				
More than 8 hours/week	37.9	1.25 (0.52–2.99)	29.1	1.29 (0.56–2.95)
>4–8 hours/week	33.9	0.99 (0.49–1.98)	15.5	0.56 (0.23–1.37)
>2–4 hours/week	33.0	1	24.4	1
>0–2 hours/week	30.7	0.90 (0.50–1.64)	28.9	1.24 (0.49–3.19)
0 hours/week	41.1	1.40 (0.69–2.82)	20.7	0.79 (0.25–2.44)

^a ^Adjusted for body mass index and smoking.

* Statistically significant association (p < 0.05)

### Nature of Activity and NSP

No statistically significant associations were found for NSP (ever, past month) and dynamic or static activities (Table 4). No significant association between chronic NSP and dynamic activities was established. In females, static activities of >130–135 hours per week and >145–155 hours per week compared to >135–145 hours per week were found to be associated with chronic NSP (OR 3.72; 95% CI 1.17–11.86, and OR 2.94; 95% CI 1.14–7.59 respectively). Time spent in sitting and standing was not found to be associated with NSP in the past month (Table 4) or chronic NSP. Lying activities of >65–70 hours per week compared to >70–80 hours per week was associated with NSP in the past month in females only (OR 3.51; 95% CI 1.80–6.87) (Table 4), while no association was established between chronic NSP and lying. NSP ever was associated with lying >65–70 hours compared to >70–80 hours in females (OR 2.13; 95% CI 1.08–4.19) and sitting >60–70 hours compared to >50–60 hours in males (OR 2.07; 95%CI 2.11–3.84). There was no significant association between NSP ever and duration of standing.

**Table 4:** Logistic regression of neck/shoulder pain (NSP) in the past month and nature of activity for females (n = 351) and males (n = 292), expressed as odds ratio and 95% confidence intervals (CI)

Nature of activity	**Females**		**Males**	
	Proportion reporting NSP (%)	Adjusted^a ^OR (CI)	Proportion reporting NSP (%)	Adjusted^a ^OR (CI)
**Dynamic**				
More than 40 hours/week	39.3	1.62 (0.70–3.72)	13.3	0.57 (0.12–2.70)
>30–40 hours/week	40.6	1.60 (0.90–2.86)	34.0	1.97 (0.96–4.04)
>20–30 hours/week	30.3	1	21.1	1
>15–20 hours/week	31.0	1.0 (0.52–1.92)	25.0	1.24 (0.55–2.79)
15 hours/week or less	47.4	2.11 (0.81–5.52)	20.4	0.97 (0.44–2.14)
				
**Static**				
More than 155 hours/week	33.3	0.97 (0.17–5.55)	16.7	0.69 (0.22–2.22)
>145–155 hours/week	33.0	0.95 (0.56–1.61)	25.0	1.13 (0.59–2.17)
>135–145 hours/week	34.0	1	22.7	1
>130–135 hours/week	34.2	1.02 (0.48–2.18)	22.6	1.01 (0.39–2.60)
130 hours/week or less	37.0	1.20 (0.60–2.40)	29.6	1.44 (0.56–3.66)
				
**Lying**				
More than 90 hours/week	38.2	1.42 (0.65–3.07)	27.6	1.13 (0.44–2.88)
>80–90 hours/week	28.0	0.82 (0.44–1.52)	17.5	0.61 (0.29–1.26)
>70–80 hours/week	30.4	1	26.0	1
>65–70 hours/week	61.2	*3.51 (1.80–6.87)	30.6	1.26 (0.59–2.66)
65 hours/week or less	20.0	0.56 (0.22–1.46)	14.8	0.49 (0.16–1.56)
				
**Sitting**				
More than 70 hours/week	29.0	0.81 (0.36–1.79)	23.5	1.37 (0.58–3.22)
>60–70 hours/week	34.8	1.02 (0.57–1.83)	24.4	1.44 (0.69–3.01)
>50–60 hours/week	34.2	1	18.3	1
>40–50 hours/week	33.9	1.02 (0.53–1.95)	29.2	1.90 (0.81–4.41)
40 hours/week or less	37.8	1.26 (0.58–2.72)	27.8	1.75 (0.54–5.68)
				
**Standing**				
More than 20 hours/week	40.5	1.13 (0.55–2.32)	33.3	2.01 (0.33–12.09)
>15–20 hours/week	32.6	0.83 (0.47–1.48)	17.9	0.85 (0.28–2.59)
>10–15 hours/week	36.4	1	20.0	1
>5–10 hours/week	30.4	0.76 (0.41–1.42)	23.5	1.21 (0.61–2.41)
5 hours/week or less	16.7	0.34 (0.07–1.61)	31.4	1.81 (0.82–4.02)

^a ^Adjusted for body mass index and smoking.

* Statistically significant association

### Type of Sedentary Activity and NSP

Time spent reading, watching television, using a computer and participating in all electronic information technology activities (computer and TV) was not found to be associated with NSP in the past month (Table 5) and chronic NSP. Watching TV >6–10 hours compared to 6 hours or less per week was found to be protective against NSP ever in males (OR 0.26; 95% CI 0.09–0.76).

**Table 5:** Logistic regression of neck and shoulder pain (NSP) in the past month and sedentary activity for females (n = 351) and males (n = 292), expressed as odds ratio and 95% confidence intervals (CI)

**Sedentary activity**	**Females**		**Males**	
	Proportion reporting NSP (%)	Adjusted^a ^OR (CI)	Proportion reporting NSP (%)	Adjusted^a ^OR (CI)
**Time watching TV**				
More than 32 hours/week	36.0	0.66 (0.23–1.87)	22.5	0.72 (0.22–2.33)
>23–32 hours/week	38.6	0.69 (0.28–1.70)	21.1	0.65 (0.22–1.94)
>10–23 hours/week	30.3	0.51 (0.25–1.05)	25.0	0.81 (0.31–2.15)
>6–10 hours/week	32.8	0.59 (0.26–1.35)	19.4	0.59 (0.17–1.97)
6 hours/week or less	47.4	1	29.2	1
				
**Total reading time**				
More than 4 hours/week	40.0	1.52 (0.24–9.80)	33.3	1.61 (0.27–9.48)
>2–4 hours/week	37.5	1.36 (0.52–3.59)	37.0	1.89 (0.75–4.81)
>1–2 hours/week	33.8	1.17 (0.57–2.38)	21.1	0.88 (0.35–2.22)
>0–1 hours/week	35.1	1.19 (0.65–2.17)	21.1	0.86 (0.45–1.65)
0 hours/week	30.7	1	23.5	1
				
**Time at computer**				
More than 12 hours/week	38.3	1.35 (0.57–3.19)	21.1	0.65 (0.23–1.83)
>6–12 hours/week	30.1	0.90 (0.39–2.09)	22.1	0.70 (0.24–1.99)
>3–6 hours/week	33.3	1.09 (0.48–2.49)	23.6	0.75 (0.26–2.21)
>0–3 hours/week	36.1	1.25 (0.55–2.82)	25.8	0.85 (0.30–2.42)
0 hours/week	33.3	1	29.2	1
				
**Total IT time (TV + computer)**				
More than 46 hours/week	18.2	0.52 (0.10–2.73)	25.5	1.28 (0.31–5.35)
>35–46 hours/week	37.8	1.45 (0.59–3.56)	21.4	1.00 (0.24–4.21)
>18–35 hours/week	33.3	1.18 (0.60–2.32)	22.1	1.05 (0.27–4.01)
>12–18 hours/week	40.0	1.60 (0.73–3.50)	31.3	1.69 (0.38–7.46)
12 hours/week or less	30.2	1	21.3	1

^a ^Adjusted for body mass index and smoking.

## Discussion

NSP is a common experience in adolescence and indeed was reported by around half of the adolescents in this Australian pregnancy cohort. Although there have been several previous cross-sectional studies conducted to explore the association between NSP and activity, these have yielded inconsistent findings. In an attempt to add robust evidence to a limited body of knowledge from sufficiently large cohort studies [26,30,31], we examined this association in a large Western Australian pregnancy cohort – the RAINE cohort. Despite rich data describing the level, nature and type of weekly physical activity, we could not identify a consistent relationship between self-reported NSP and physical activity registered in the last week. Given our comprehensive data, we suggest that NSP is *not *related to physical activity in adolescents and that the significant associations reported here between NSP and physical activity are most likely due to chance.

The prevalence of NSP reported in this study is broadly consistent with other international datasets, reporting the 1 month prevalence to range from 11–34% [31,34,48,49]. Although, Diepenmaat et al [31] reported a lower 1 month prevalence of NSP (11.5%) in a cohort of Dutch adolescents aged 12–16 years, this is likely attributable to a more stringent definition of pain in their study (pain for ≥ 4 days in the month) and inclusion of younger adolescents. Females reported a higher prevalence of NSP across all prevalence periods, consistent with previous literature and other musculoskeletal pain experiences in adults [50], adolescents [31,34,51,52] and children [15,53]. Although physical activity behaviours differ somewhat between males and females separate gender analysis of the NSP-physical activity relationships did not yield consistent gender differences. A higher prevalence of self-reported pain among females may be due to differences in musculoskeletal maturity, posture, endocrine, and psychosocial characteristics as well as different physiological mechanisms for pain perception between genders [54].

The intensity level and nature of weekly physical activity were not consistently related to the prevalence of NSP in a dose-response manner. Random significant findings of an association between NSP and intensity and nature of physical activity are likely due to chance from multiple comparisons. Although previous research has established an association between NSP and vigorous physical activity [15], this relationship was only for traumatic musculoskeletal pain and not for non-traumatic pain. Similarly, an earlier cohort study reported that a high level of physical activity was related to 6 month prevalence of NSP in adolescents who experienced pain at least once per week [30]. Although we reported a similar finding, that females who engaged in more than 8 hours of vigorous physical activity were 8 times more likely to report chronic NSP, the confidence interval for this estimate was very large and sample size low for this category, increasing the risk a chance finding. The absence of an association between NSP in the last month and vigorous physical activity should also be considered in the context of confounding factors such as the analgesic effect of endorphin release after vigorous physical activity [55], and that adolescents who participate in vigorous physical activity are stronger and therefore have a musculoskeletal system more resilient to pain. Similarly, the absence of an association between sedentary activity, for example IT use, and NSP in the last month might be influenced by adolescents with a history of pain learning to use IT more appropriately.

Our results are consistent with other reports in the literature where no clear relationship was established between NSP and non-sedentary activities We examined in detail the association between the nature of physical activity and NSP given that different physical activities impart very different biomechanical and psychological stresses, raising the possibility that some activities are beneficial while others harmful to spinal health [34]. There is also evidence that exposure time to certain physical activities increases the risk of developing spinal pain [56], yet despite this rationale, we could not identify an association between physical activity type and intensity, and NSP. Other investigators have also found no association between neck pain and the duration and intensity of physical activity [14,15,26,31,32]. Mogensen et al [34] investigated the relationship between spinal pain and the type and duration of sport among 439 adolescents aged 12–13 years. Consistent with our data, they did not find an association between NSP and the type or duration of sport in general. However, one month prevalence of neck pain was positively associated with horse-riding and negatively associated with soccer. However, these findings should be interpreted with some caution considering the small sample size in each sport (n = 33 for horse-riding and n = 136 for soccer) and the possibility of selection bias. Nonetheless, the finding suggests that sport-specific characteristics impart unique effects on the spine. Niemi et al [29] reported that sporting activities which loaded the upper limbs had a protective effect on NSP in females, while Cardon et al [16] reported that females with NSP engaged less often in moderate physical activity. We did not find any association between one month prevalence of NSP and dynamic activities and vigorous activity and this may be due to a lack of specificity in the domains.

The exposure to various physical and sedentary activities in this Australian pregnancy cohort was very similar to a Finnish birth cohort [26]. However, Australian adolescents tended to spend a greater amount of time watching television, less time reading, and had a broader spread of individuals across the time intervals for computer use. The weekly volume of sedentary activity was not related to NSP in the last month or chronic NSP, consistent with an earlier study examining the volume of television watching and computer use [31]. However, this is in contrast to Auvinen et al [26] who reported significant associations between neck-occipital and shoulder pain (females only) with sitting time in a dose-response manner. Specific sedentary activities associated with neck-occipital pain were watching television and reading for females, playing or working at the computer for males, and time spent in other sedentary activities for both genders. In contrast, shoulder pain was only associated with watching 4 or more hours of television and 2 or more hours of other sedentary activities in females. There are several reasons why our results differ to those of Auvinen et al [26]. Firstly, we collected a combined measure of neck and shoulder pain. Considering the fewer associations between inactivity and shoulder pain reported by Auvinen et al [26], our data may have lacked specificity for neck pain, or sedentary activities may be more related to upper cervical (occipital) pain. For example, poor upper cervical posture or a forward-head posture is a common clinical observation in patients with upper cervical spine symptoms. Secondly, the recall period differed between studies (one month vs. 6 months). Thirdly, the mode of data collection for physical activity was different between studies. Finally, the adolescents in the Finnish cohort were aged 16 years while the mean age of our Australian cohort was 14.0 years and NSP prevalence is known to increase with age through adolescence [13,14,29,30,57]. For example, at age 18, the prevalence of NSP is at least double that at age 14 [30]. Although other studies have reported that adolescent NSP is positively related to not participating in any physical activity [28,30], interpreting these findings is difficult since the nature of physical inactivity was not described in detail.

The current study has a number of strengths. The advantage of MARCA as a tool to collect physical activity information over traditional questionnaires is that it provides a richer data source regarding the intensity, duration, and nature of all diurnal physical activities over a one week period. In contrast, Auvinen et al [26] collected physical activity data via questionnaire only with respect to activities outside school and mode of commuting to/from school. Whereas MARCA assigns a MET value to physical activities [40], the Finnish adolescents were required to self-report 'brisk' or 'light' intensity. Furthermore, MARCA collects total day physical activity information across the continuum of physical activity – from very sedentary activities to very vigorous activities. MARCA week day and weekend day data was collected over a three year period and therefore includes seasonal and vacation-related variability in physical activity habits. Secondly, we have utilised data from a large birth cohort which provides a large sample size and validity in generalising findings to the population. To our knowledge there are only 3 other large European population-based cohort studies examining this topic [26,30,31]. We also adjusted for important covariates, conducted separate analyses for males and females and allowed for different relationships with different levels of activity.

Limitations of this study should also be considered. Physical activity data were self-reported and collected over a brief time period. Although there is concern regarding the agreement between self-reports and objective measures of physical activity [35], MARCA data does correlate more strongly than questionnaire data with accelerometry and more accurate estimates of energy expenditure may be generated [39,58]. Moreover, objective measures cannot capture the nature of physical activity well nor the type of sedentary activity. The prevalence of NSP was self-reported which may also introduce responder bias. Nonetheless, the Nordic Musculoskeletal Pain Questionnaire agrees with an objective clinical assessment [59]. We performed a large number of statistical computations which is likely to increase the chance of type 1 error. Given the cross-sectional design of this study, we cannot infer any causation, merely association. Consequently, we are unable to judge whether activity characteristics are mediated by NSP experiences. It may be that adolescents who have experienced NSP avoid those activities which propagate NSP, thereby eliminating any association in a cross-sectional analysis. Therefore, it will be important to prospectively investigate the association between NSP and physical activity and consider other factors which mediate physical activity in order to confidently determine the risk of physical activity behaviours for NSP. Prospective data suggest that the natural course of neck pain fluctuates over childhood, yet for a small subgroup of children with concurrent musculoskeletal symptoms and psychologic distress, neck pain persists [60]. It may be that a more detailed examination of physical activity patterns in this subgroup would clarify the association between neck pain and physical activity. In addition to exploring the association between NSP and physical activity in subgroups with particular comorbidities, the association should also be explored in subgroups of adolescents based on postural profiles where unique and clinically important loading patterns are likely to mediate the relationship [61].

## Conclusion

The relationship between self-reported NSP and physical activity in adolescents has been unclear due to inconsistencies among reports. These inconsistencies have largely been attributable to variability in operational definitions of pain and measurement of physical activity and sedentary activity. Despite comprehensive data describing physical activity patterns we were unable to identify a consistent relationship between self-reported one month and lifetime NSP prevalence and physical activity measured in five minute blocks registered over a one week period in Australian adolescents. Therefore, we conclude that self-reported one month and lifetime NSP prevalence in adolescents is not related to the level or intensity of physical activity or the type of sedentary activity over a one week period. Whether physical activity characteristics are related to the development or persistence of NSP should be investigated prospectively while considering factors that mediate physical activity behaviour.

## Competing interests

The authors declare that they have no competing interests.

## Authors' contributions

AB contributed to the design of the study, project management, data analysis, and manuscript writing. NB was responsible for data analysis and contributed to manuscript writing. AS and LS contributed to the design of the study, project management, data analysis, and manuscript writing. All authors read and approved the final version of the manuscript.

## Authors' information

AB is an NHMRC postdoctoral research fellow and clinical physiotherapist. LS is a senior NHMRC research fellow. NB is a clinical paediatric physiotherapist and research fellow. AS is an NHMRC postdoctoral research fellow.

## Pre-publication history

The pre-publication history for this paper can be accessed here:



## References

[B1] Hogg-Johnson S, Velde G van der, Carroll LJ, Holm LW, Cassidy JD, Guzman J, Cote P, Haldeman S, Ammendolia C, Carragee E, Hurwitz E, Nordin M, Peloso P (2008). The burden and determinants of neck pain in the general population. Results of the bone and joint decade 2000–2010 task force on neck pain and its associated disorders. Spine.

[B2] Cote P, Kristman V, Vidmar M, Van Eerd D, Hogg-Johnson S, Beaton D, Smith PM (2008). The prevalence and incidence of work absenteeism involving neck pain. A cohort of Ontario lost-time claimants. Spine.

[B3] Ariens GAM, Bongers PM, Hoogendoorn WE, Houtman ILD, Wal G van der, van Mechelen W (2001). High quantitative job demands and low coworker support as risk factors for neck pain – Results of a prospective cohort study. Spine.

[B4] Ariens GAM, van Mechelen W, Bongers PM, Bouter LM, Wal G van der (2000). Physical risk factors for neck pain. Scand J Work Environ Health.

[B5] Jeffries LJ, Milanese SF, Grimmer-Somers KA (2007). Epidemiology of adolescent spinal pain. A systematic overview of the research literature. Spine.

[B6] Hakala P, Rimpela A, Salminen JJ, Virtanen SM, Rimpela M (2002). Back, neck and shoulder pain in Finnish adolescents: National cross sectional surveys. Brit Med J.

[B7] Brattberg G (2004). Do pain problems in young school children persist into early adulthood? A 13-year follow-up. Eur J Pain.

[B8] Hestbaek L, leboeuf-Yde C, Kyvik KO, Manniche C (2006). The course of low back pain from adolescence to adulthood. Eight year follow-up of 9600 twins. Spine.

[B9] Jones GT, Macfarlane GJ (2005). Epidemiology of low back pain in children and adolescents. Arch Dis Childhood.

[B10] Bostrom M, Dellve L, Thomee S, Hagberg M (2008). Risk factors for generally reduced productivity – A prospective cohort study of young adults with neck or upper-extremity musculoskeletal symptoms. Scand J Work Environ Health.

[B11] Mikkelsson M (1999). One year outcome of preadolescents with fibromyalgia. J Rheum.

[B12] Mikkelsson M, Salminen JJ, Kautiainen H (1997). Non-specific musculoskeletal pain in preadolescents. Prevalence and 1-year persistence. Pain.

[B13] Siivola SM, Levoska S, Latvala K, Hoskio E, Vanharanta H, Keinanen-Kiukaanniemi S (2004). Predictive factors for neck and shoulder pain: a longitudinal study in young adults. Spine.

[B14] Kujala UM, Taimela S, Viljanen T (1999). Leisure physical activity and various pain symptoms among adolescents. Brit J Sports Med.

[B15] El-Metwally A, Salminen JJ, Auvinen A, Macfarlane G, Mikkelsson M (2007). Risk factors for development of non-specific musculoskeletal pain in preteens and early adolescents: a prospective 1-year follow-up study. BMC Musculoskel Disord.

[B16] Cardon G, De Bourdeaudhuij I, De Clercq D, Philippaerts R, Verstraete S, Geldhof E (2004). Physical fitness, physical activity, and self-reported back and neck pain in elementary schoolchildren. Ped Exercise Sci.

[B17] El-Metwally A, Salminen JJ, Auvinen A, Kautiainen H, Mikkelsson M (2004). Prognosis of non-specific musculoskeletal pain in preadolescents: A prospective 4-year follow-up study till adolescence. Pain.

[B18] Carroll LJ, Eric LH, Cote P, Hogg-Johnson S, Carragee EJ, Nordin M, Holm LW, Velde G van der, Cassidy JD, Guzman J, Peloso PM, Haldeman S (2008). Research priorities and methodological implications – The bone and joint decade 2000–2010 task force on neck pain and its associated disorders. Spine.

[B19] Carroll LJ, Hogg-Johnson S, Velde G van der, Haldeman S, Holm LW, Carragee EJ, Hurwitz EL, Cote P, Nordin M, Peloso PM, Guzman J, Cassidy JD (2008). Course and prognostic factors for neck pain in the general population – Results of the bone and joint decade 2000–2010 task force on neck pain and its associated disorders. Spine.

[B20] Briggs AM, Straker LM, Wark JD (2009). Bone health and back pain: What do we know and where should we go?. Osteoporos Int.

[B21] Dollman J, Norton K, Norton L (2005). Evidence for secular trends in children's physical activity behaviour. Brit J Sports Med.

[B22] Marshall SJ, Biddle SJH, Gorely T, Cameron N, Murdey I (2004). Relationships between media use, body fatness and physical activity in children and youth: A meta-analysis. Int J Obesity.

[B23] Magarey AM, Daniels LA, Boulton TJ (2001). Prevalence of overweight and obesity in Australian children and adolescents: Reassessment of the 1985 and 1995 data against new standard international definitions. Med J Aust.

[B24] Matthiessen J, Groth MV, Fagt S, Biltoft-Jensen A, Stockmarr A, Andersen JS, Trolle E (2008). Prevalence and trends in overweight and obesity among children and adolescents in Denmark. Scand J Public Health.

[B25] Kautiainen S, Rimpela A, Vikat A, Virtanen SM (2002). Secular trends in overweight and obesity among Finnish adolescents in 1977–1999. Int J Obesity.

[B26] Auvinen J, Tammelin T, Taimela S, Zitting P, Karppinen J (2007). Neck and shoulder pains in relation to physical activity and sedentary activities in adolescence. Spine.

[B27] Holth HS, Werpen HKB, Zwart JA, Hagen K (2008). Physical inactivity is associated with chronic musculoskeletal complaints 11 years later: Results from the Nord-Trondelag Health Study. BMC Musculoskel Disord.

[B28] Alricsson M, Landstad BJ, Romild U, Werner S (2006). Self-related health, physical activity and complaints in Swedish high school students. The Scientific World Journal.

[B29] Niemi S, Levoska S, Kemila J, Rekola K, Keinanen-Kiukaanniemi S (1996). Neck and shoulder symptoms and leisure time activities in high school students. J Orthop Sports Phys Ther.

[B30] Vikat A, Rimpela M, Salminen JJ, Rimpela A, Savolainen A, Virtanen SM (2000). Neck or shoulder pain and low back pain in Finnish adolescents. Scand Journal Public Health.

[B31] Diepenmaat ACM, Wal MF van der, de Vet HCW, Hirasing RA (2006). Neck/shoulder, low back, and arm pain in relation to computer use, physical activity, stress, and depression among Dutch adolescents. Pediatrics.

[B32] Ehrmann Feldman D, Shrier I, Rossignol M, Abenhaim L (2002). Risk factors for the development of neck and upper limb pain in adolescents. Spine.

[B33] Mikkelsson LO, Nupponen H, Kaprio J, Kautiainen H, Mikkelsson M, Kujala UM (2006). Adolescent flexibility, endurance strength, and physical activity as predictors of adult tension neck, low back pain, and knee injury: A 25 year follow up study. Br J Sports Med.

[B34] Mogensen AM, Gausel AM, Wedderkopp N, Kjaer P, Leboeuf-Yde C (2007). Is active participation in specific sport activities linked with back pain?. Scand J Med Sci Sports.

[B35] Wedderkopp N, Leboeuf-Yde C, Bo Andersen L, Froberg K, Steen Hansen H (2003). Back pain in children: No association with objectively measured level of physical activity. Spine.

[B36] Kendall GE (2003). Children in families in communities: A modified conceptual framework and an analytic strategy for identifying patterns of factors associated with developmental health problems in childhood. PhD thesis.

[B37] Kuorinka I, Jonsson B, Kilbom A, Vinterverg H, Biering-Sorensen F, Andersson G, Jorgensen K (1987). Standardised Nordic questionnaires for the analysis of musculoskeletal symptoms. Appl Ergon.

[B38] Dickinson CE, Campion K, Foster AF, Newman SJ, O'Rourke AMT, Thomas PG (1992). Questionnaire development: An examination of the Nordic Musculoskeletal Questionnaire. Appl Ergon.

[B39] Ridley K, Olds TS, Hill A (2006). The multimedia activity recall for children and adolescents (MARCA): development and evaluation. Int J Behav Nutr and Phys Act.

[B40] Ridley K, Ainsworth BE, Olds TS (2008). Development of a compendium of energy expenditures for youth. Int J Behav Nutr and Phys Act.

[B41] Ainsworth BE, Haskell WL, Whitt MC, Irwin ML, Swartz AM, Strath SJ, O'Brien WL, Bassett DRJ, Schmitz KH, Emplaincourt PO, Jacobs DRJ, Leon AS (2000). Compendium of physical activities: An update of activity codes and MET intensities. Med Sci Sports Exerc.

[B42] Cole TJ, Bellizzi MC, Flegal KM, Dietz WH (2000). Establishing a standard definition for child overweight and obesity worldwide: International survey. Brit Med J.

[B43] Walker-Bone K, Reading I, Coggon D, Cooper C, Palmer KT (2004). The anatomical pattern and determinants of pain in the neck and upper limbs: an epidemiologic study. Pain.

[B44] Palmer KT, Syddall H, Cooper C, Coggon D (2003). Smoking and musculoskeletal disorders: findings from a British national survey. Ann Rheum Dis.

[B45] Makela M, Heliovaara M, Sievers K, Impivaara O, Knekt P, Aromaa A (1991). Prevalence, determinants, and consequences of chronic neck pain in Finland. Amer J Epidemiol.

[B46] Kjaer P (2004). Low back pain in relation to lumbar spine abnormalities as identified by magnetic resonance imaging. PhD thesis.

[B47] Dionne CE, Dunn KM, Croft PR, Nachemson AL, Buchbinder R, Walker BF, Wyatt M, Cassidy JD, Rossignol M, Leboeuf-Yde C, Hartvigsen J, Leino-Arjas P, Latza U, Reis S, del Real MTG, Kovacs FM, Oberg B, Cedraschi C, Bouter LM, Koes BW, Picavet HSJ, van Tulder MW, Burton K, Foster NE, Macfarlane GJ, Thomas E, Underwood M, Waddell G, Shekelle P, Volinn E, Von Korff M (2008). A consensus approach toward the standardization of back pain definitions for use in prevalence studies. Spine.

[B48] Murphy S, Buckle P, Stubbs D (2004). Classroom posture and self-reported back and neck pain in schoolchildren. Appl Ergon.

[B49] Murphy S, Buckle P, Stubbs D (2007). A cross-sectional study of self-reported back and neck pain among English schoolchildren and associated physical and psychological risk factors. Appl Ergon.

[B50] Wijnhoven HA, de Vet HCW, Picavet HSJ (2006). Prevalence of musculoskeletal disorders is systematically higher in women than in men. Clin J Pain.

[B51] Adamson G, Murphy S, Shevlin M, Buckle P, Stubbs D (2007). Profiling schoolchildren in pain and associated demographic and behavioural factors: A latent class approach. Pain.

[B52] Kovacs FM, Gestoso M, del Real MTG, Lopez J, Mufraggi N, Mendez JI (2003). Risk factors for non-specific low back pain in schoolchildren and their parents: a population based study. Pain.

[B53] De Inocencio J (2004). Epidemiology of musculoskeletal pain in primary care. Arch Dis Childhood.

[B54] LeResche L, Crombie IK, Croft PR, Linton SJ, LeResche L, von Korff M (1999). Gender considerations in the epidemiology of chronic pain. Epidemiology of Pain.

[B55] Koltyn KF, Garvin AW, Gardiner RL, Nelson TF (1996). Perception of pain following aerobic exercise. Med Sci Sports Exercise.

[B56] McMeeken J, Tully E, Stillman B, Nattrass C, Bygott IL, Story I (2001). The experience of back pain in young Australians. Man Ther.

[B57] Stahl M, Mikkelsson M, Kautiainen H, Hakkinen A, Ylinen J, Salminen JJ (2004). Neck pain in adolescence: A 4-year follow-up of pain-free preadolescents. Pain.

[B58] Ridley K, Dollman J, Olds T (2001). Development and validation of a computer delivered physical activity questionnaire (CDPAQ) for children. Ped Exercise Sci.

[B59] Bjorksten MG, Boquist B, Talback M, Edling C (1999). The validity of reported musculoskeletal problems. A study of questionnaire answers in relation to diagnosed disorders and perception of pain. Appl Ergon.

[B60] Stahl M, Kautiainen H, El-Metwally A, Hakkinen A, Ylinen J, Salminen JJ, Mikkelsson M (2008). Non-specific neck pain in schoolchildren: Prognosis and risk factors for occurrence and persistence. A 4-year follow-up study. Pain.

[B61] Smith A, O'Sullivan P, Straker L (2008). Classification of sagittal thoraco-lumbo-pelvic alignment of the adolescent spine in standing and its relationship to low back pain. Spine.

